# Supplementary material for: Depletion of Gibberellin Signaling Up-Regulates LBD16 Transcription and Promotes Adventitious Root Formation in Arabidopsis Leaf Explants
Source: Int J Mol Sci. 2024 Dec 12;25(24):13340. doi: 10.3390/ijms252413340 (PMC11678481; doi:10.3390/ijms252413340)
Supplement: Supplementary file 1 [file ijms-25-13340-s001.zip › ijms-3287384-supplementary.pptx]

## Slide 1
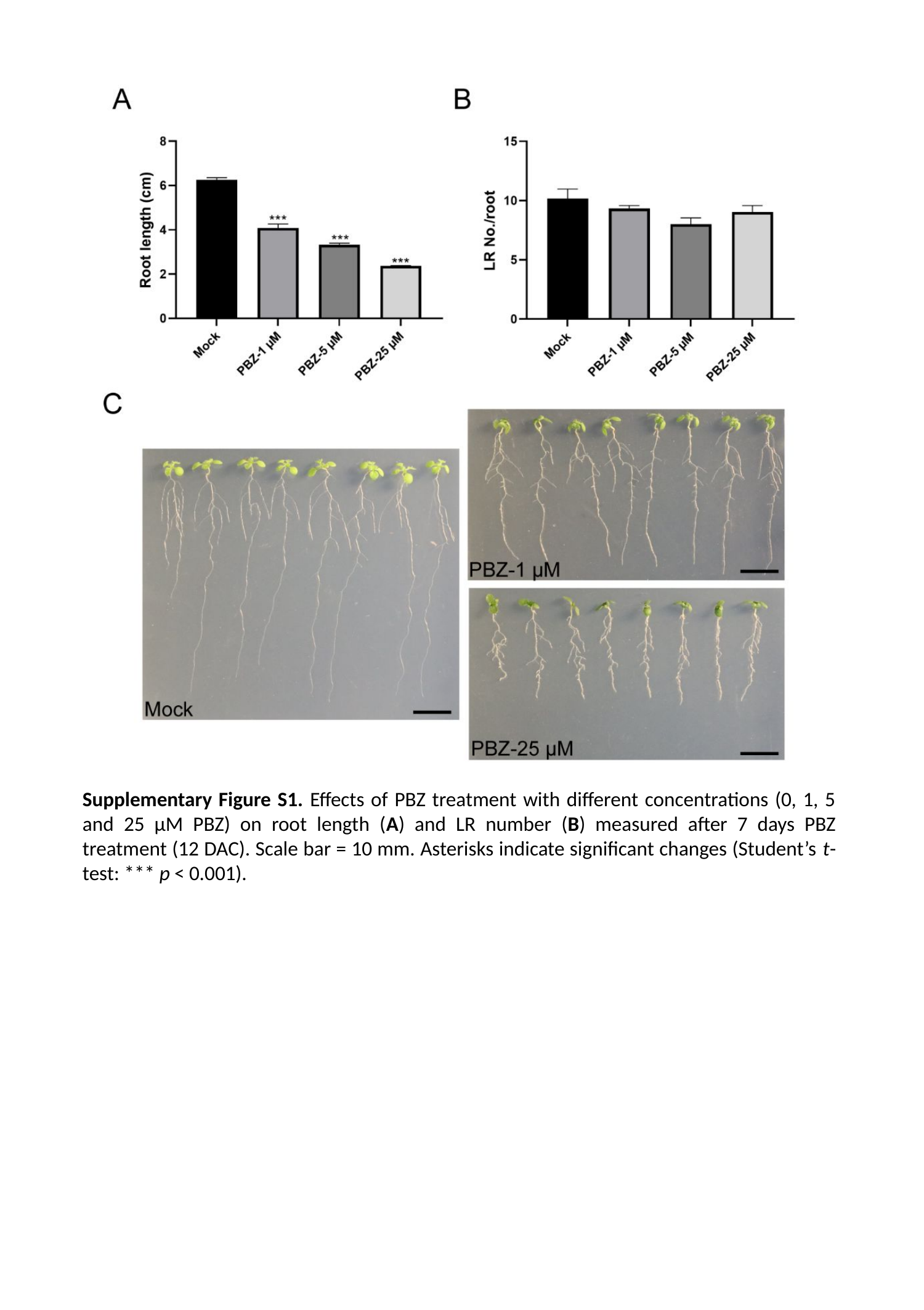

Supplementary Figure S1. Effects of PBZ treatment with different concentrations (0, 1, 5 and 25 µM PBZ) on root length (A) and LR number (B) measured after 7 days PBZ treatment (12 DAC). Scale bar = 10 mm. Asterisks indicate significant changes (Student’s t-test: *** p < 0.001).

## Slide 2
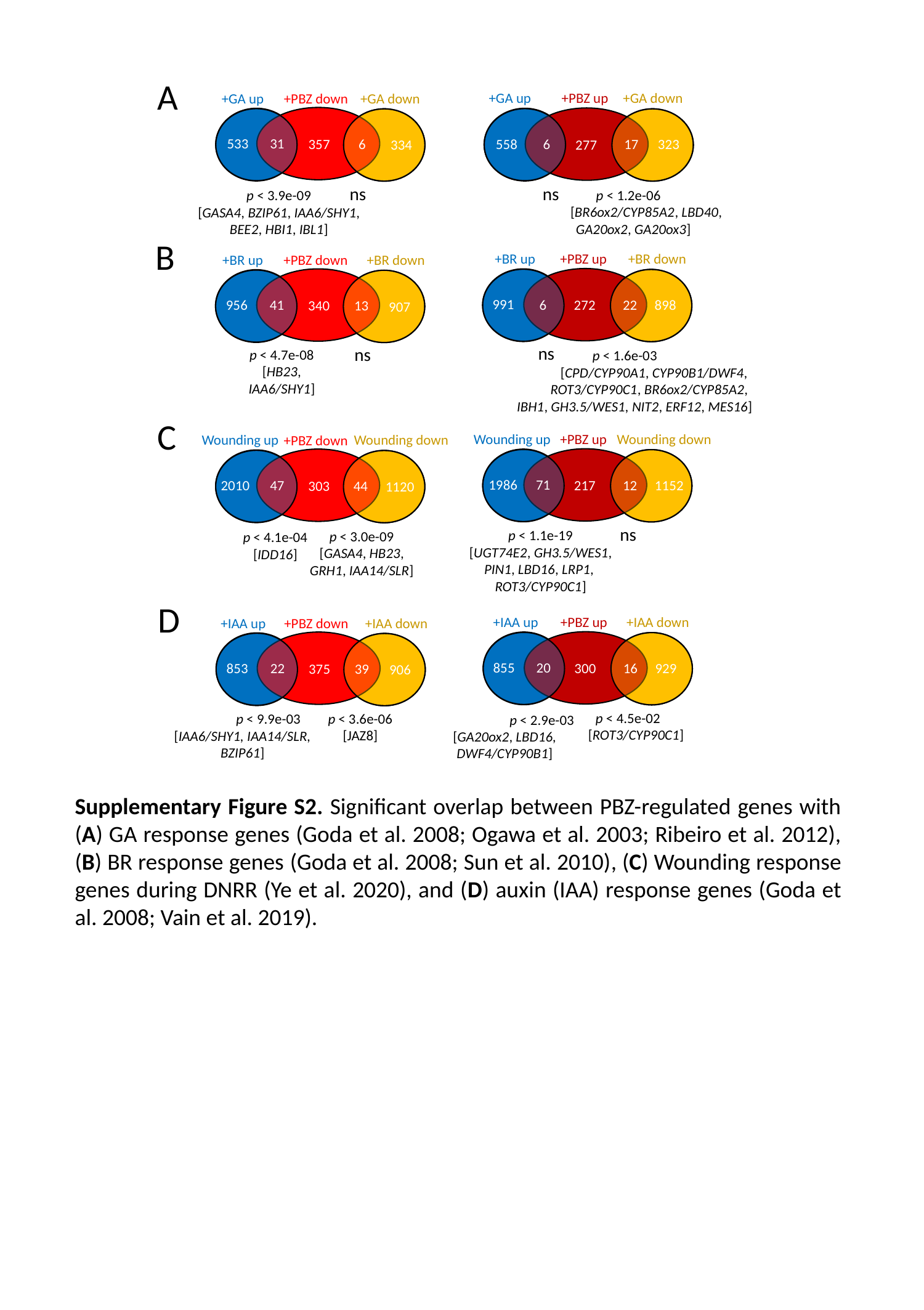

A
+PBZ up
+GA up
+GA down
+GA up
+PBZ down
+GA down
31
533
558
6
6
357
323
17
277
334
ns
ns
p < 1.2e-06
 [BR6ox2/CYP85A2, LBD40,
 GA20ox2, GA20ox3]
p < 3.9e-09
[GASA4, BZIP61, IAA6/SHY1,
BEE2, HBI1, IBL1]
B
+BR up
+BR down
+PBZ up
+BR up
+BR down
+PBZ down
991
6
898
22
272
41
956
13
340
907
ns
ns
p < 4.7e-08
[HB23,
IAA6/SHY1]
p < 1.6e-03
 [CPD/CYP90A1, CYP90B1/DWF4,
 ROT3/CYP90C1, BR6ox2/CYP85A2,
 IBH1, GH3.5/WES1, NIT2, ERF12, MES16]
C
Wounding up
Wounding down
+PBZ up
Wounding up
Wounding down
+PBZ down
1986
71
1152
12
217
47
2010
44
303
1120
ns
p < 1.1e-19
[UGT74E2, GH3.5/WES1,
PIN1, LBD16, LRP1,
ROT3/CYP90C1]
p < 3.0e-09
[GASA4, HB23,
GRH1, IAA14/SLR]
p < 4.1e-04
[IDD16]
D
+IAA up
+IAA down
+PBZ up
+IAA up
+IAA down
+PBZ down
855
20
929
16
300
22
853
39
375
906
p < 4.5e-02
[ROT3/CYP90C1]
 p < 9.9e-03
[IAA6/SHY1, IAA14/SLR,
BZIP61]
p < 3.6e-06
[JAZ8]
 p < 2.9e-03
[GA20ox2, LBD16,
DWF4/CYP90B1]
Supplementary Figure S2. Significant overlap between PBZ-regulated genes with (A) GA response genes (Goda et al. 2008; Ogawa et al. 2003; Ribeiro et al. 2012), (B) BR response genes (Goda et al. 2008; Sun et al. 2010), (C) Wounding response genes during DNRR (Ye et al. 2020), and (D) auxin (IAA) response genes (Goda et al. 2008; Vain et al. 2019).

## Slide 3
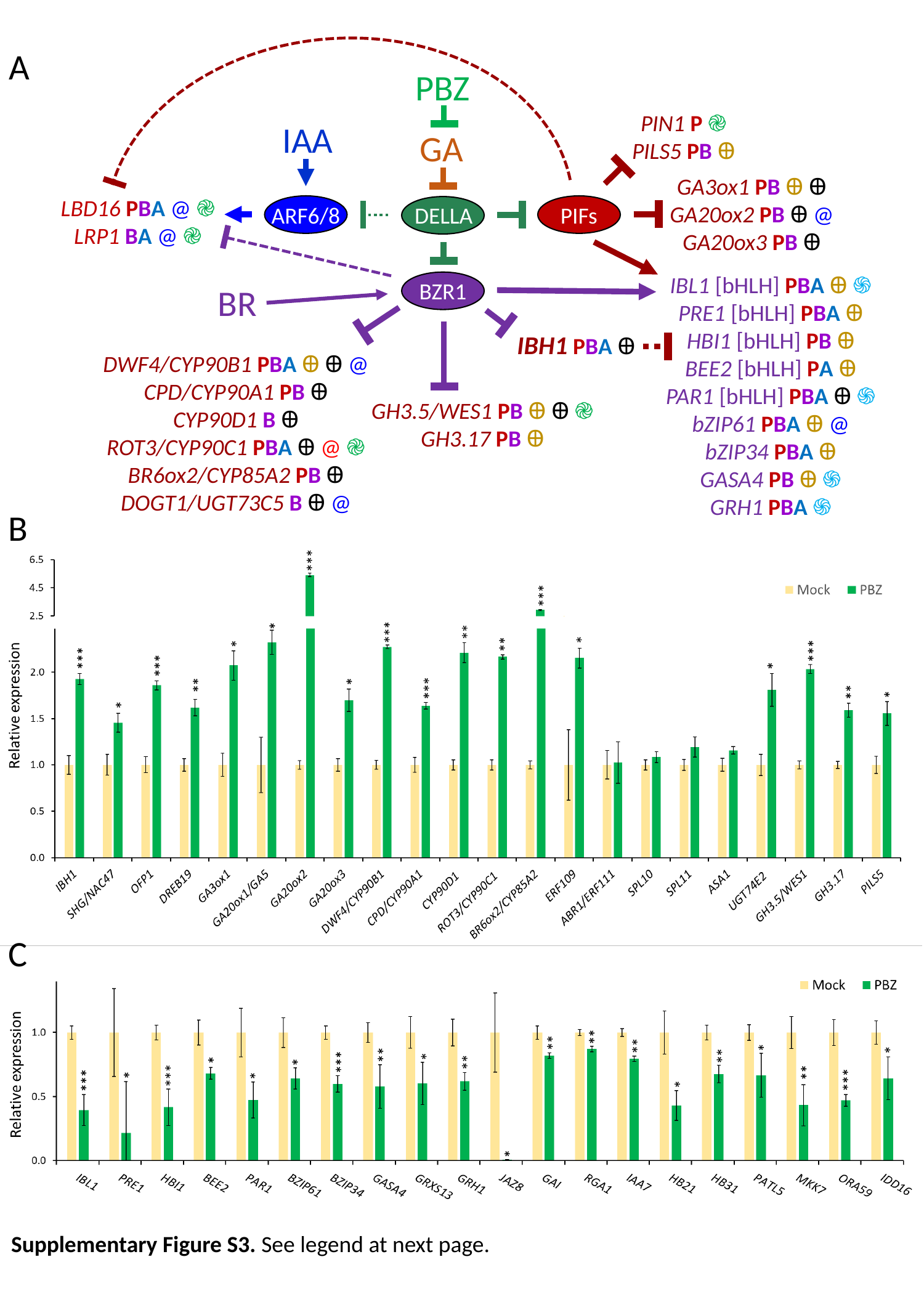

PBZ
PIN1 P ֎
PILS5 PB Ꚛ
IAA
GA
GA3ox1 PB Ꚛ Ꚛ
GA20ox2 PB Ꚛ @
GA20ox3 PB Ꚛ
LBD16 PBA @ ֎
LRP1 BA @ ֎
ARF6/8
PIFs
DELLA
IBL1 [bHLH] PBA Ꚛ ֍
PRE1 [bHLH] PBA Ꚛ
HBI1 [bHLH] PB Ꚛ
BEE2 [bHLH] PA Ꚛ
PAR1 [bHLH] PBA Ꚛ ֍
bZIP61 PBA Ꚛ @
bZIP34 PBA Ꚛ
GASA4 PB Ꚛ ֍
GRH1 PBA ֍
BZR1
BR
IBH1 PBA Ꚛ
DWF4/CYP90B1 PBA Ꚛ Ꚛ @
CPD/CYP90A1 PB Ꚛ
CYP90D1 B Ꚛ
ROT3/CYP90C1 PBA Ꚛ @ ֎
BR6ox2/CYP85A2 PB Ꚛ
DOGT1/UGT73C5 B Ꚛ @
GH3.5/WES1 PB Ꚛ Ꚛ ֎
GH3.17 PB Ꚛ
A
B
C
Supplementary Figure S3. See legend at next page.

## Slide 4
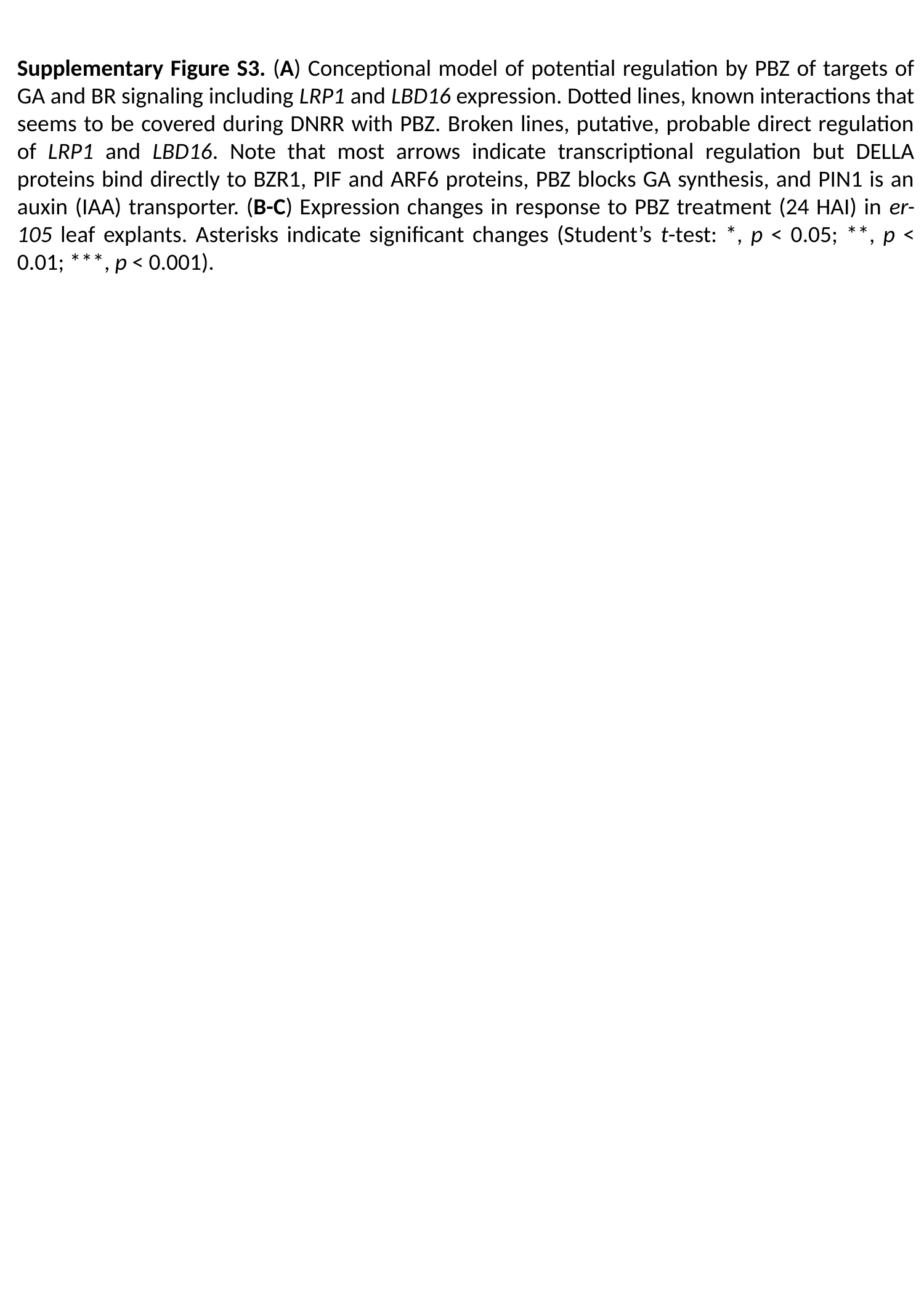

Supplementary Figure S3. (A) Conceptional model of potential regulation by PBZ of targets of GA and BR signaling including LRP1 and LBD16 expression. Dotted lines, known interactions that seems to be covered during DNRR with PBZ. Broken lines, putative, probable direct regulation of LRP1 and LBD16. Note that most arrows indicate transcriptional regulation but DELLA proteins bind directly to BZR1, PIF and ARF6 proteins, PBZ blocks GA synthesis, and PIN1 is an auxin (IAA) transporter. (B-C) Expression changes in response to PBZ treatment (24 HAI) in er-105 leaf explants. Asterisks indicate significant changes (Student’s t-test: *, p < 0.05; **, p < 0.01; ***, p < 0.001).

## Slide 5
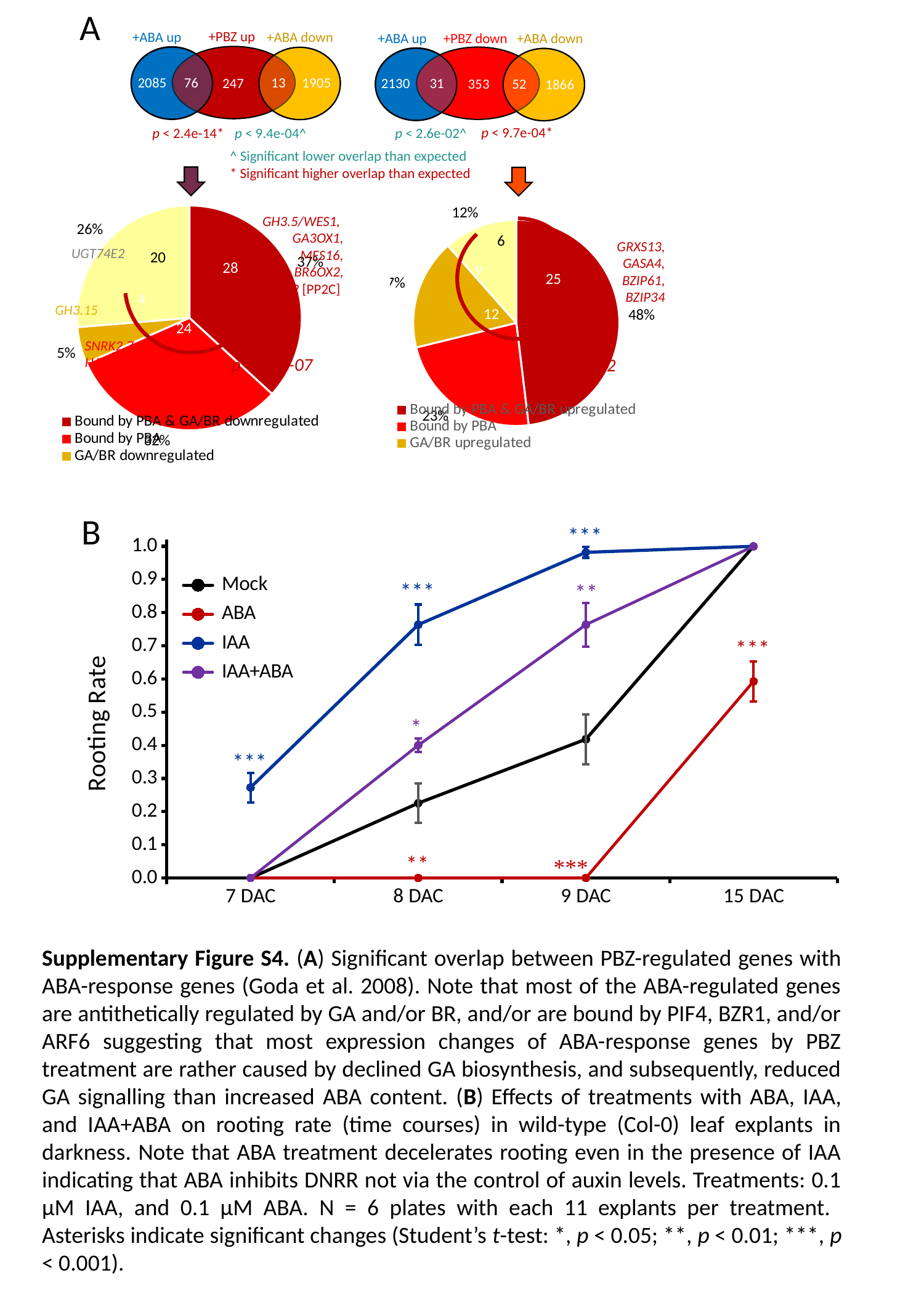

A
+PBZ up
+ABA up
+ABA down
+ABA up
+PBZ down
+ABA down
2085
76
1905
13
247
31
2130
52
353
1866
p < 9.7e-04*
p < 2.6e-02^
p < 2.4e-14*
p < 9.4e-04^
^ Significant lower overlap than expected
* Significant higher overlap than expected
### Chart
| Category | |
|---|---|
| Bound by PBA & GA/BR upregulated | 25.0 |
| Bound by PBA | 12.0 |
| GA/BR upregulated | 9.0 |
| none | 6.0 |
### Chart
| Category | |
|---|---|
| Bound by PBA & GA/BR downregulated | 28.0 |
| Bound by PBA | 24.0 |
| GA/BR downregulated | 4.0 |
| none | 20.0 |GH3.5/WES1,
GA3OX1,
MES16,
BR6OX2,
HAI2 [PP2C]
6
GRXS13,
GASA4,
 BZIP61,
BZIP34
UGT74E2
20
28
9
25
4
GH3.15
12
24
74%
p < 2.9e-07
88%
p < 2.1e-12
SNRK2.7
HAI1 [PP2C]
GRH1
B
### Chart
| Category | Mock | ABA | IAA | IAA+ABA |
|---|---|---|---|---|
| 7 DAC | 0.0 | 0.0 | 0.2727272727272727 | 0.0 |
| 8 DAC | 0.2254545454545455 | 0.0 | 0.7636363636363637 | 0.4 |
| 9 DAC | 0.41818181818181815 | 0.0 | 0.9818181818181818 | 0.7636363636363637 |
| 15 DAC | 1.0 | 0.5927272727272727 | 1.0 | 1.0 |Supplementary Figure S4. (A) Significant overlap between PBZ-regulated genes with ABA-response genes (Goda et al. 2008). Note that most of the ABA-regulated genes are antithetically regulated by GA and/or BR, and/or are bound by PIF4, BZR1, and/or ARF6 suggesting that most expression changes of ABA-response genes by PBZ treatment are rather caused by declined GA biosynthesis, and subsequently, reduced GA signalling than increased ABA content. (B) Effects of treatments with ABA, IAA, and IAA+ABA on rooting rate (time courses) in wild-type (Col-0) leaf explants in darkness. Note that ABA treatment decelerates rooting even in the presence of IAA indicating that ABA inhibits DNRR not via the control of auxin levels. Treatments: 0.1 µM IAA, and 0.1 µM ABA. N = 6 plates with each 11 explants per treatment. Asterisks indicate significant changes (Student’s t-test: *, p < 0.05; **, p < 0.01; ***, p < 0.001).

## Slide 6
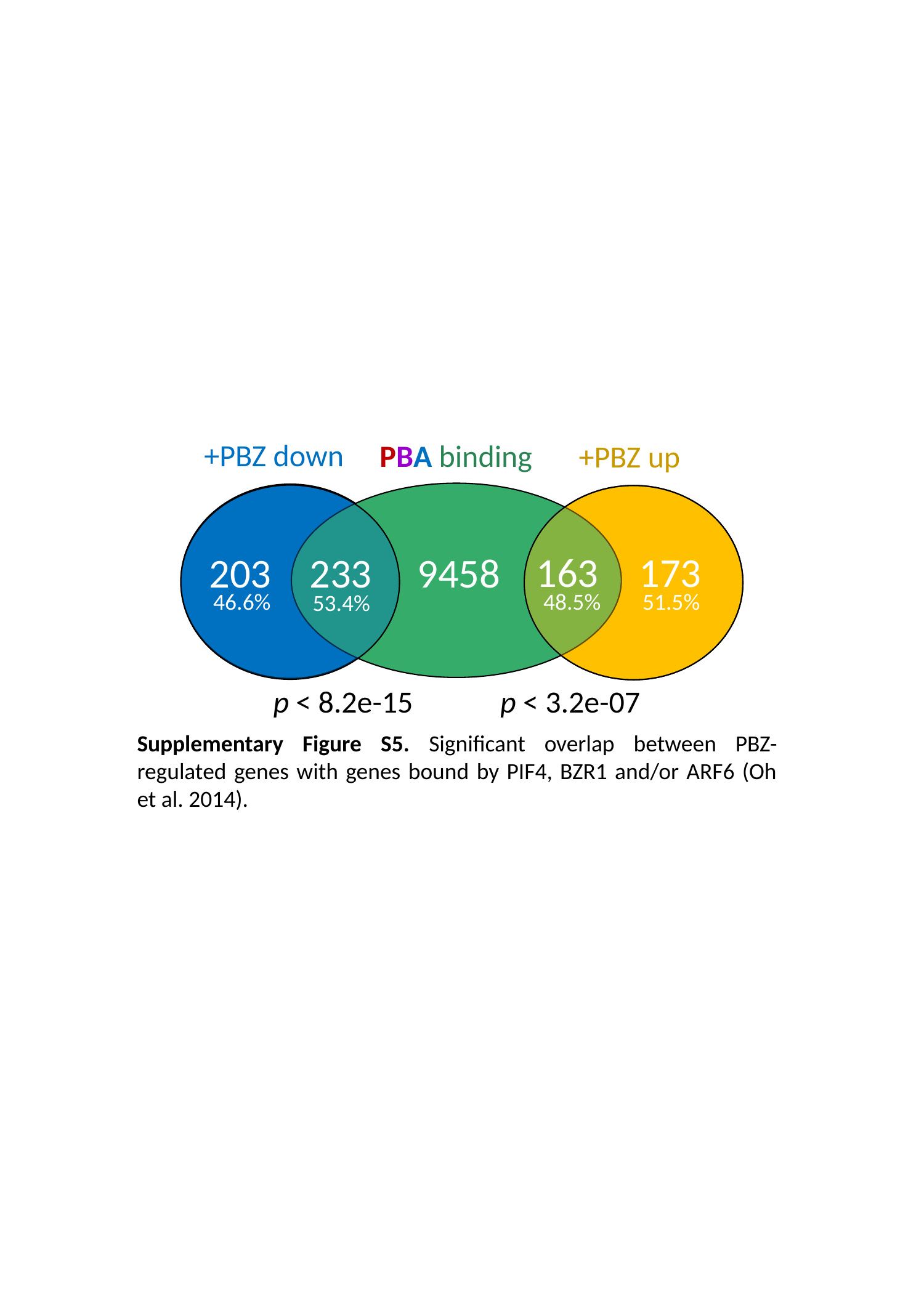

+PBZ down
PBA binding
+PBZ up
173
163
203
233
9458
48.5%
51.5%
46.6%
53.4%
p < 3.2e-07
p < 8.2e-15
Supplementary Figure S5. Significant overlap between PBZ-regulated genes with genes bound by PIF4, BZR1 and/or ARF6 (Oh et al. 2014).
